# Study on Sulfate Migration Behavior of Potassium Magnesium Phosphate Cement Slurry Based on Electro-Pulse-Accelerated Corrosion

**DOI:** 10.3390/ma18225158

**Published:** 2025-11-13

**Authors:** De Xu, Qing Yang, Jianming Yang, Xuexing Hu

**Affiliations:** 1 School of Architectural Engineering, Jiangsu Open University, Nanjing 210036, China; xude@jsou.edu.cn (D.X.); yangq@jsou.edu.cn (Q.Y.); 2 Research Center of Intelligent Construction and Blockchain Collaborative Innovation, Jiangsu Open University, Nanjing 210036, China; 3 College of Civil Engineering, YanCheng Institute of Technology, Yancheng 224051, China; 19852106031@163.com

**Keywords:** potassium magnesium phosphate cement (PMPC), sulfate attack behavior, electro-pulse-accelerated sulfate corrosion, SO_4_^2−^ migration law, SO_4_^2−^ migration coefficient, nickel slag powder, silica fume

## Abstract

By accelerating the migration of sulfate ions in potassium magnesium phosphate cement (PMPC) paste through an electric field, its sulfate resistance can be quickly evaluated, thereby making up for the defect of long test cycles in existing evaluation methods. Through sulfate concentration analysis, strength tests, microanalysis and theoretical analysis, this paper investigated the SO_4_^2−^ migration behavior of PMPC specimens subjected to electro-pulse-accelerated corrosion. The conclusions are as follows: the distribution of SO_4_^2−^ concentration c (x, t) in PMPC specimens followed a polynomial pattern with corrosion period t. The surface SO_4_^2−^ concentration c (0, t), measured SO_4_^2−^ migration depth h_0_, and c (x, t) of specimens increased with the t. After 56 days, the c (0, 56 days) and h_0_ of the PN containing nickel slag powder and the PS containing silica fume were lower than that of the reference P0. Their calculated SO_4_^2−^ migration depth h_00_ and SO_4_^2−^ migration coefficient D were smaller than that of P0. The h_00_ and D could be estimated based on t due to a logarithmic relationship between t and h_00_, D. The strength of specimens at the pulse cathode end gradually improved with t. The 56-day strength for P0, PN, and PS specimens increased by 7.14%, 7.94%, and 8.42%, respectively. The research findings provided a theoretical foundation for the application and quality evaluation of PMPC-based material.

## 1. Introduction

Portland cement concrete is prone to chemical erosion from harmful components in air, groundwater, and soil due to hydration products and its porous structure, leading to a decline in its strength and service life. Among various types of chemical corrosion, sulfate erosion is the most complex and harmful type of environmental water erosion [1,2]. Magnesium phosphate cement (MPC), composed of dead-burned magnesia, soluble phosphates, and additives, compared to Portland cement, has characteristics such as rapid setting, high early strength, strong adhesion, and low pH [3,4]. The hardened MPC system has a dense ceramic-like structure that can hinder the invasion of harmful ions. The hardened body mainly consists of struvite (or K-struvite) and unreacted dead-burned magnesia, which has good stability in neutral and weakly alkaline environments (pH = 7~11) [3] and strong resistance to salt corrosion, and has been studied for applications in repair and anti-corrosion coatings [4,5,6,7,8]. In the MPC system, potassium magnesium phosphate cement (PMPC) uses potassium dihydrogen phosphate (KDP) as the acid component. KDP is produced from the thermal processing of phosphate rock, where the residual risk of radioactive elements is significantly lower than that of ammonium dihydrogen phosphate (ADP) produced from the wet processing of phosphate rock [9,10]. PMPC has received more attention due to its relatively controllable hydration rate and the absence of polluting gases during construction [5,6,7,8].

In actual engineering, sulfate attack on concrete is a slow physicochemical reaction process. The resistance of concrete to sulfate attack is generally evaluated using accelerated test methods [11]. According to the Chinese standard, ‘Method for Testing Resistance of Cement to Sulfate Attack’ (GB/T749-2008) [12], and the American standard, ‘Standard Test Method for Potential Expansion of Portland Cement Mortar Exposed to Sulfate’ (ASTM C452) [13], different specimen sizes of 10 mm × 10 mm × 30 mm and 25 mm × 25 mm × 285 mm are used, respectively. Due to the small size of the specimens, differences in the manufacturing process, erosion environment conditions, and testing conditions may significantly affect the test results, leading to high variability in parallel test results. The Chinese standard, ‘Standard Test Methods for Long-term Performance and Durability of Ordinary Concrete’ (GB/T50082-2009) [14], uses a method that combines increased temperature and concentration of the eroding solution with dry–wet cycles. The dry–wet cycles more closely simulate the characteristics of sulfate attack on concrete structures in actual engineering. Increasing the temperature of the eroding solution accelerates the diffusion rate of SO_4_^2−^, thus accelerating the sulfate attack on concrete, but it also increases the solubility of various hydration products and causes changes in the composition and structure of the hydration products, thereby altering the mechanism of sulfate attack on concrete and affecting the assessment of its resistance to sulfate attack. Based on electrokinetic principles, charged ions in the solution can obtain greater acceleration from the electric field, significantly increasing the migration speed of ‘hot’ charged ions. If a pulse voltage is applied to cement concrete specimens immersed in a sulfate solution, an electric potential gradient will form at the ends of the capillaries in the water-saturated specimens, accelerating the migration of eroding ions. Yang and Chang et al. [15,16] used an electric field to accelerate the penetration of chloride ions into cement-based materials, studied methods to enhance the chloride ion resistance of cement-based materials, and achieved success. Sylvie et al. [17] studied the transport of sulfates within concrete under both free diffusion and electric field conditions. The study showed that the depth to which sulfates penetrate concrete under an electric field is much greater than under free diffusion. When specimens were immersed and electrified for 6 months, the strength loss under free diffusion was relatively small, whereas the strength decreased significantly under electrified conditions. Wang et al. [18,19,20] studied the sulfate attack on cement concrete under electric pulse action at room temperature. The research showed that electric pulses accelerate the penetration of SO_4_^2−^ ions into the interior of cement-based materials, with the best effect at the cathode of the specimen. It also accelerates the chemical reaction between SO_4_^2−^ ions and cement-based materials. This indicates that the method of accelerating the sulfate resistance of concrete based on the principle of electric pulses is technically feasible, providing a basis for the development of electric pulses as an acceleration technology for the sulfate resistance of cement-based materials.

Previous studies [21,22,23,24] have used accelerated corrosion methods, such as sulfate immersion, sulfate dry–wet cycles, and sulfate freeze–thaw cycles, to research and evaluate the SO_4_^2−^ resistance behavior of MPC systems, confirming that their sulfate resistance is superior to that of ordinary Portland cement concrete. However, these methods have drawbacks, such as long experimental periods or significant differences from natural corrosion environments. Researchers assess the degree of sulfate attack on MPC via the macroscopic damage levels of the eroded MPC paste (strength loss, deformation, and mass loss), which fails to reflect the migration of sulfate ions within the hardened body. When using MPC systems in a high SO_4_^2−^-corrosive condition, the migration of sulfate ions within the hardened body is a prerequisite for the progression of the corrosion reaction and the occurrence of damage. To deeply understand the sulfate attack mechanism in MPC systems, it is necessary to start by studying the SO_4_^2−^ migration [25]. The project team investigated the SO_4_^2−^ migration law in PMPC paste soaked in a 5% Na_2_SO_4_ for 360 days, summarizing the SO_4_^2−^ migration mechanisms in the PMPC system and establishing corresponding migration models [26,27,28,29], but the experimental period was too long. Referring to existing studies [28,29,30], this paper accelerated the corrosion of PMPC paste specimens through the application of electric pulses. By comparing the sulfate ion diffusion behaviors of different PMPC paste specimens, the sulfate resistance of the PMPC paste was evaluated. The research findings provide a theoretical foundation for the application and quality evaluation of PMPC-based material.

## 2. Materials and Methods

### 2.1. Materials

Dead-burned magnesia powder (MP) is obtained from magnesite through high-temperature electric melting at 1500 °C, followed by ball milling, with an average particle size of 45.26 μm, provided by Huanren Oriental Red Hydropower Station Magnesia Sand Factory, Hai Cheng, Liaoning, China. Nickel slag powder (NSP) is sourced from Jiangsu Sanding Metal Co., Ltd. (Wuxi, Jiangsu, China), with an average particle size of 38.71 μm. Silica fume (SF) is provided by Luoyang Yumin Silica Micro Powder Co., Ltd. (Luoyang, Henan, China), with a particle size range of 0.01~0.5 μm. The main components of MgO powder, nickel slag powder, and silica fume are listed in Table 1. The phosphate used is a food-grade potassium dihydrogen phosphate (KDP, with a KH_2_PO_4_ mass fraction ≥ 98%), having a main particle size range of 40/350~60/245 (mesh/μm), and is supplied by Lianyungang Gelichemical Co., Ltd. (Lianyungang, Jiangsu, China). The new composite retarding agent (NCR) consists of borax, sodium diphosphate decahydrate, and magnesium chloride hexahydrate in a mass ratio of 1:2:1; all these components are analytical reagents.

### 2.2. Slurry and Specimen Preparation

**Figure 1:**
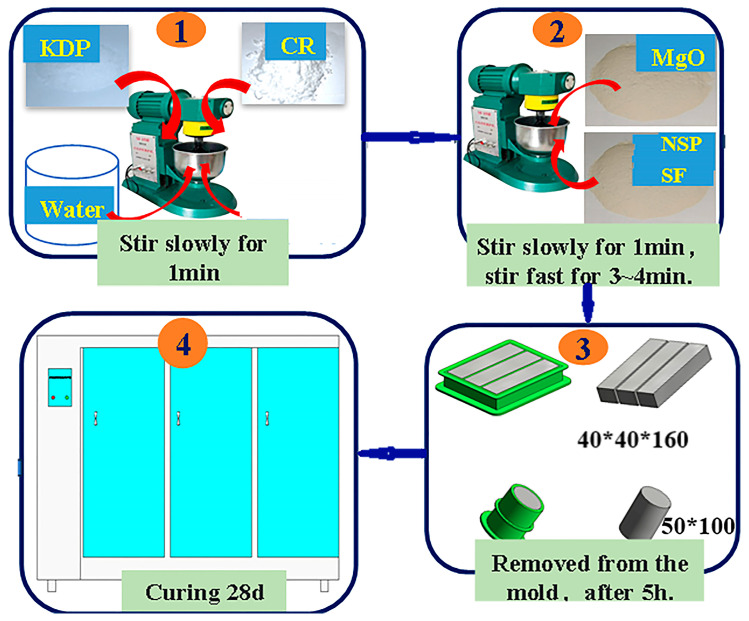
Preparation of PMPC slurry and specimens.

Referring to previous research findings [21,22,23], this study used the mix ratio shown in Table 2. The environmental temperature was maintained at 22–25 °C. According to the mix ratio in Table 2, the acid, base, retard and water were weighed. Following the sequence in Figure 1(①,②), KDP and CR were first poured into a mixing pot of the NJ-160A type cement paste mixer and 50% of the water was added, with slow stirring for 1 min (57–67 r/min). MgO powder, NSP/SF, and the other 50% of the water were added into the mixing pot, with slow stirring for 1 min and then with quick stirring (115–135 r/min) for 3–4 min. Then, the stirring was stopped and the slurry was scraped off on the blade. Finally, the PMPC slurry was obtained. The slurry was poured into molds (see Figure 1(③)); a 40 mm × 40 mm × 160 mm prism specimen was used to test strength and a 50 mm diameter × 100 mm length cylindrical specimen was used to test the concentration of SO_4_^2−^. The slurry was vibrated to compact and level it. After an initial setting, the slurry specimen was covered with cling film. After 5 h, the specimens were placed in a curing room with a temperature of 20–22 °C and a relative humidity of 60–80% for curing until 28 days (Figure 1(④)).

### 2.3. Test Methods

#### 2.3.1. Macro Performance Test

**Figure 2:**
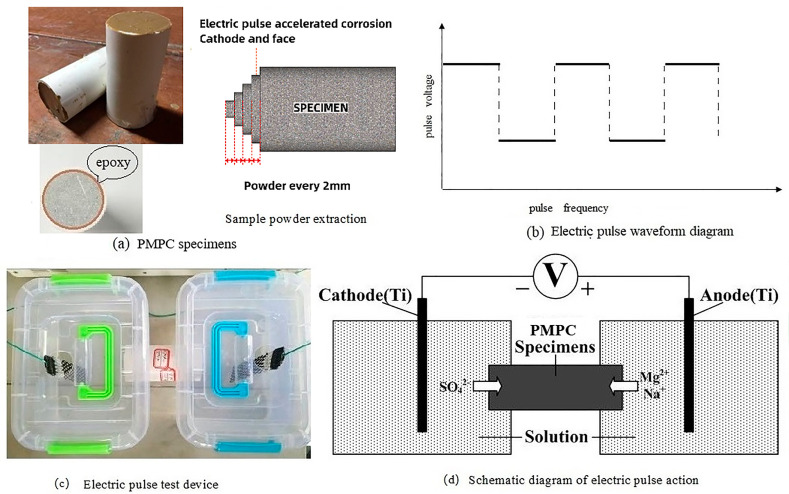
Electric pulse accelerated corrosion test.

According to reference [31], use the NLD-S-type cement mortar flow tester manufactured by Wuxi Jianyi Instruments Co., Ltd. (Wuxi, Jiangsu, China) to test the fluidity of the PMPC paste. According to reference [32], use a universal testing machine (WED-300) to test the strength of the specimens, where the loading speed for flexural load is 50 N/s ± 10 N/s and the loading speed for compressive load is 2400 N/s ± 200 N/s. Due to the effect of the electric field, sulfate ions continuously move from the cathode to the anode of the specimen (as shown in Figure 2d), resulting in a gradient distribution of sulfate ions within the specimen. The PMPC specimens subjected to electro-pulse corrosion are divided into two halves at the midpoint, and the cylindrical specimens of the negative electrode terminal are used to test the change in the compressive strength of the specimen after electro-pulse corrosion.

#### 2.3.2. Sulfate Corrosion Test

When the hydration age reached 28 days, the PMPC paste specimens were subjected to sulfate corrosion tests. The epoxy resin was used to seal the contact area between the PMPC specimens and the PVC pipe to satisfy the one-dimensional transport of the SO_4_^2−^ (see Figure 2a). Before the sulfate corrosion test, the specimens were placed in a vacuum saturation tank to fully saturate them with the solution. Based on existing research results [27,30], the electro-pulse corrosion regime was as follows: 5% MgSO_4_ for the anode solution, 5% Na_2_SO_4_ for the cathode solution, pulse voltage of 60 V, pulse frequency of 30 s (15 s on, 15 s off), and a DC stabilized power supply was used to apply the electro-pulse, with the waveform shown in Figure 2b. The contact area between the outer mold of the specimen and the container was sealed with epoxy to prevent the solution from seeping out of the joints (see Figure 2c). Titanium metal mesh was chosen as the electrode and was connected to the pulse power source, with the electrodes placed in the two ends of the erosion solutions (see Figure 2c). The pH values of the corrosion solutions at both ends were regularly tested, using a pH meter, during the corrosion process. A schematic diagram of the principle of accelerated corrosion testing using electro-pulse is shown in Figure 2d.

#### 2.3.3. Sulfate Concentration Analysis

When the corrosion age was reached, we removed the PMPC specimens with PVC molds that had been accelerated by electro-pulse solution corrosion, demolded them, and cleaned them thoroughly. We placed them in an oven at 50–60 °C until they reached a constant weight. Samples were taken by slicing the specimen’s cathode end face (see Figure 2a). The powders were obtained by slicing through a 150-mesh sieve, and we referred to references [28,29] to analyze the content of sulfate ions in the powder samples.

#### 2.3.4. Microanalysis

Samples were taken from specimens with 28-day hydration ages and subjected to electrical pulses for 56 days (2 mm away from the cathode end). Small block samples and powder samples (finer than 150 mesh) were soaked in anhydrous ethanol (to terminate hydration). During the analysis, the PMPC samples were first removed and dried to a constant weight. Microscopic analysis of the PMPC samples was conducted according to the requirements in Table 3.

## 3. Test and Analysis Results

### 3.1. The Migration Law of Sulfate Ions in the PMPC Slurry Specimens

**Figure 3:**
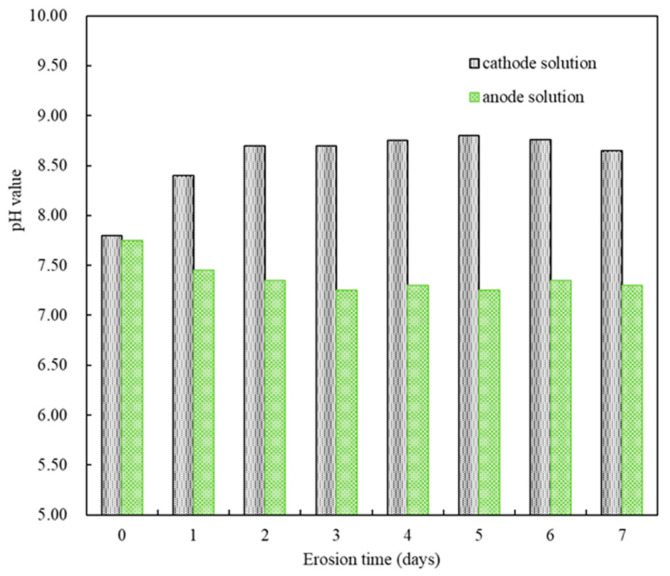
The pH changes in the solution at both electrodes.

Figure 3 displayed the pH changes in the sulfate solution at both electrodes during accelerated corrosion for seven days under the set pulse regime. In the first two days of applying the electric pulse, the pH of the sulfate solution (5% MgSO_4_) at the cathode gradually increased from an initial 7.80 to 8.70, with bubbles observed in the solution. From day three to day seven of the pulse, the pH of the sulfate solution fluctuated between 8.60 and 8.80. Conversely, in the first two days of applying the electric pulse, the pH of the sulfate solution (5% Na_2_SO_4_) at the anode decreased from an initial 7.75 to 7.35, with bubbles also observed. From day three to day seven of the pulse, the pH of the sulfate solution fluctuated between 7.25 and 7.35. Considering that the pH values of the solutions at both electrodes had stabilized from day three to day seven after the start of the electric pulse, and referring to existing research results [27,30], the experiment was set to change the corrosion solution every seven days to replenish the consumed SO_4_^2−^ ions and prevent acid corrosion caused by accumulated H^+^ ions. The basic test and analysis cycle was set to 14 days of electric pulses.

**Figure 4:**
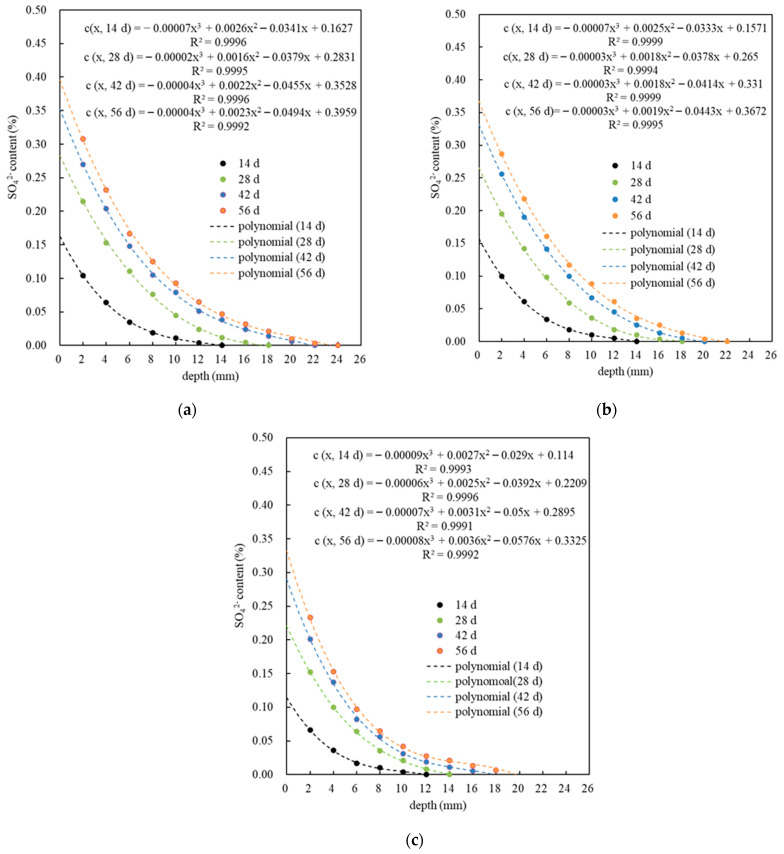
The migration law of the SO_4_^2−^ in PMPC specimens: (**a**) P0; (**b**) PN; and (**c**) PS.

Figure 4a–c shows the distribution of sulfate ion content *c* (*x*, *t*) in PMPC specimens after accelerated corrosion for different corrosion ages under a set pulse current regime. The distribution patterns of the sulfate ion contents in different PMPC specimens are basically consistent: the *c* (*x*, *t*) in PMPC specimens with corrosion age (*t*) decreased rapidly with increasing distance from the surface (*x*), and followed a third-order polynomial rule under the condition of correlation coefficient *R*^2^ ≥ 0.999. As the pulse period increased, the surface sulfate content *c* (0, *t*), the measured sulfate erosion depth *h*_0_, and the *c* (*x*, *t*) at the same erosion depth all increased gradually. When the pulse period was 14 days, the c (0, 14 d) and h_0_ of P0, PN, and PS were 0.1627%, 0.1571%, and 0.1140% and 14 mm, 14 mm, and 12 mm, respectively. When the pulse period was 56 days, the c (0, 56 d) and h_0_ of P0, PN, and PS were 0.3959%, 0.3672%, and 0.3325% and 24 mm, 22 mm, and 20 mm, respectively. The results indicated that a moderate amount of NSP or SF could significantly strengthen the SO_4_^2−^ diffusion resistance of the PMPC paste. Compared with existing research [28,29], the sulfate migration speed and migration depth is significantly greater than that of fully immersed PMPC specimens, which provides an experimental verification for the rapid evaluation of the sulfate resistance of PMPC-based materials by applying electric pulses.

### 3.2. Calculation of Sulfate Ion Migration Parameters in PMPC Specimens

Based on the fitting polynomials in Figure 4a–c and the measured sulfate ion migration depths (*h*_0_) of the PMPC specimens, the calculated SO_4_^2−^ migration depth *h*_00_ in the PMPC test pieces (Table 4) can be calculated. The *h_00_* values for different PMPC test pieces at various corrosion ages are shown in Table 4 (obtained using trial methods [28,29]). Using Fick’s second diffusion law (Laplace transform and its inverse transform), the migration parameters *c* (*0, t*), *c* (*h*_00_*, t*), and *h*_00_ can be calculated for each corrosion age t in Table 4. The sulfate ion diffusion coefficient *D* in the PMPC specimens can be calculated along with the Gaussian error function table. The results are shown in Table 4. Table 4 shows that the *h*_00_ values at all corrosion ages were greater than the *h*_0_ for specimens P0, PN, and PS. After 56 days of electro-pulse corrosion, the *h*_00_ values for specimens P0, PN, and PS were 21 mm, 20 mm, and 17 mm, respectively. For specimens P0, PN, and PS, subjected to electro-pulse corrosion, the diffusion coefficient *D* was highest at a corrosion age of 14 days, and then gradually decreased with increasing corrosion age. The *D* for specimens P0, PN, and PS reached 6.18 × 10^−6^, 4.78 × 10^−6^, and 3.42 × 10^−6^, respectively, at 56-day corrosion ages, and were one order of magnitude higher than those of fully immersed PMPC specimens after 360 days of corrosion [28,29]. The results indicated that electro-pulse treatment significantly accelerated the migration of sulfate ions in PMPC specimens; an appropriate amount of NSP or SF can significantly decrease the migration parameters, *h*_00_ and *D*, of the PMPC specimens.

### 3.3. Evaluation of the Sulfate Resistance of PMPC Slurry

**Figure 5:**
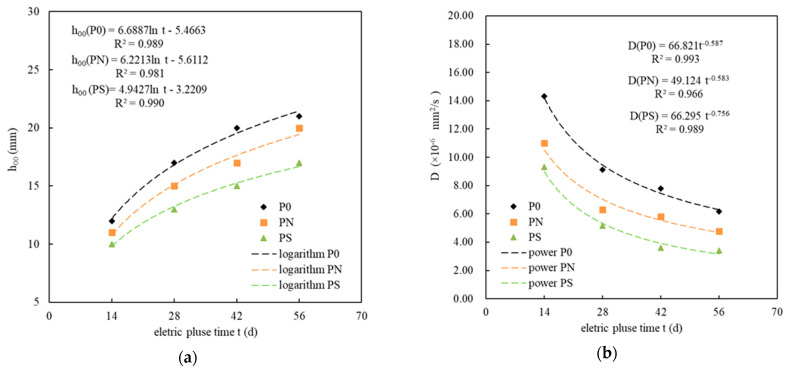
The relationship between the age of sulfate pulse corrosion and the calculated migration depth, with the migration coefficient: (**a**) the relationship between *h*_00_ and *t*; (**b**) the relationship between *D* and *t*.

Figure 5a shows the relationship between the corrosion age *t* and the calculated sulfate migration depth *h*_00_ of the PMPC specimens subjected to pulse corrosion. For the specimens P0, PN, and PS, subjected to pulse corrosion, *t* and *h*_00_ followed a logarithmic relationship, and their calculation models and correlation coefficients are listed in Table 5, where R_2_ > 0.98. The sulfate migration depth *h*_00_ could be estimated based on the corrosion age *t*. Figure 5b illustrates the relationship between the corrosion age *t* and the sulfate migration coefficient *D* of the PMPC specimens subjected to pulse corrosion. For the specimens P0, PN, and PS, subjected to pulse corrosion, *t* and *D* followed a power function relationship, and their calculation models and correlation coefficients are listed in Table 5, where R_2_ > 0.98. The sulfate migration coefficient *D* could be estimated based on the corrosion age *t*. Therefore, the resistance of PMPC specimens to sulfate attack could be evaluated through short-term accelerated corrosion tests using electric pulses.

### 3.4. The Fluidity and Strength of PMPC Slurry Specimens

Table 2 shows the fluidity and strength of different PMPC slurries. The NSP and SF replaces MgO powder in an amount of 20% and 10%, and the water–cement ratio is appropriately adjusted to ensure comparable fluidity. As shown in Table 2, an appropriate amount of NSP or FS can improve the fluidity of the PMPC slurry, thereby reducing the water–cement ratio of the PMPC paste with basically the same fluidity. An appropriate amount of NSP or FS can improve the strengths of the PMPC slurry specimens. At 28 days of hydration age, the flexural and compressive strengths of the PN specimen containing nickel slag powder and the PS specimen containing silica fume were increased by 4.0% and 6.6%, 5.6%, and 12.2%, respectively, compared to reference P0.

**Figure 6:**
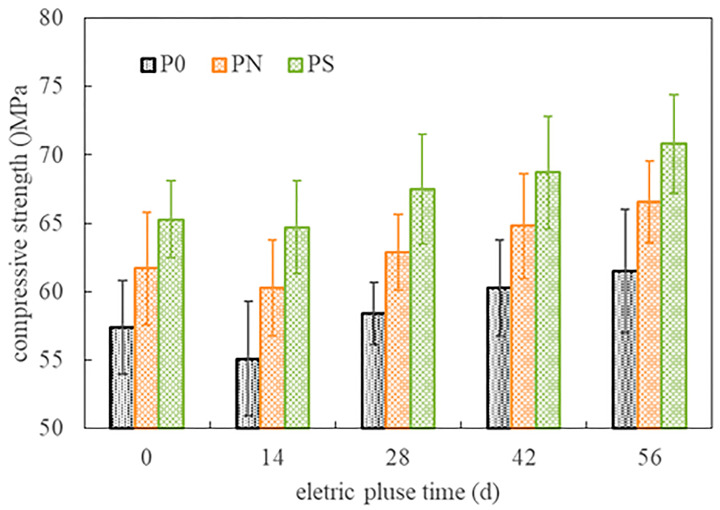
The compressive strength of cylindrical specimens (negative electrode end).

Figure 6 shows the compressive strength development of the cylindrical specimens of the pulsed negative terminal at different ages during pulse corrosion. When the corrosion age was zero, the compressive strength of the cylindrical specimen was the strength after being saturated in the vacuum-saturated water tank with the same solution (close to the 28-day strength in Table 2). The order of strength, from low to high, was P0, PN, and PS. After fourteen days of pulse corrosion, the compressive strength of the specimens was slightly lower than the initial strength, with decreases of 4.01%, 2.27%, and 1.84% for specimens P0, PN, and PS, respectively. As the corrosion age extended, the strength of the PMPC specimens gradually increased. After fifty-six days of pulse corrosion, the increases in strength for specimens P0, PN, and PS were 7.14%, 7.94%, and 8.42%, respectively.

### 3.5. Phase Composition and Pore Structure Analysis

**Figure 7:**
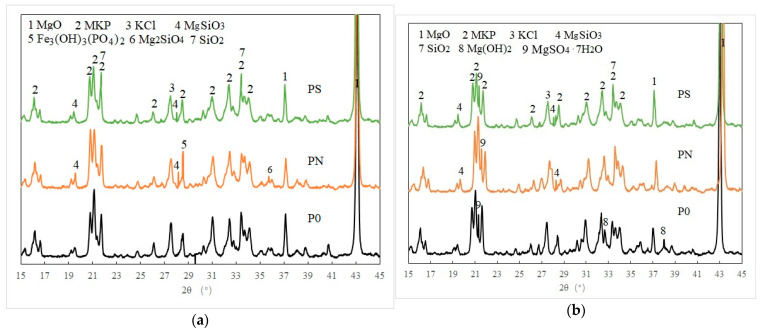
XRD spectrum of PMPC samples: (**a**) 28 d hydration ages; (**b**) 56 d pulse ages.

Phase analysis of PMPC samples subjected to hydration for 28 days and electrochemical corrosion for 56 days was conducted using XRD, with results shown in Figure 7. In Figure 7a, the main diffraction peaks of samples P0, PN, and PS after 28 days of hydration are with MKP and unreacted MgO. A characteristic peak of KCl crystal appears at 2θ ≈ 26.5–27.7°, which is likely due to the combination of Cl^−^ from CR with K^+^ from KDP. Multiple diffraction peaks of MgSiO_3_ are detected at 2θ ≈ 19.5–19.6° and 2θ ≈ 28.5–28.6° in samples PN and PS, indicating that the weakly alkaline and exothermic hydration environment activated the active components of NSP and SF in PN and PS specimens, which combined with Mg^2+^ and OH^−^ to form MgSiO_3_ gel, filling the pores and cracks of the PMPC slurry. A characteristic peak of Fe_3_(OH)_3_(PO_4_)_2_ is observed at 2θ ≈ 28.6° in the PN sample, suggesting that part of the iron oxide in NSP combined with phosphate to form iron phosphate. A characteristic peak of Mg_2_SiO_4_ crystals is found at 2θ ≈ 36.0° in the PN sample, while a characteristic peak of SiO_2_ is observed at 2θ ≈ 26.7° in the PS sample, which likely originates from the raw materials NSP and SF.

In the PMPC samples after 56 days of electric pulse treatment (Figure 7b), the main characteristic peaks of MKP, unreacted MgO, and KCl were also present. Additionally, the PMPC samples showed characteristic peaks of MgSO_4_·7H_2_O, which likely formed when SO_4_^2−^ migrated into the sample pores from the cathode end and the Mg^2+^ hydrolyzed via unreacted MgO in the pore solution reached saturation concentration, leading to the precipitation of MgSO_4_·7H_2_O. In the P0 sample after 56 days of electric pulse treatment, characteristic peaks of Mg(OH)_2_ were observed, which likely formed when OH^−^ migrated into the pores of PMPC samples from the cathode end, reacted with Mg^2+^ hydrolyzed via unreacted MgO, and reached saturation concentration, leading to the precipitation of Mg(OH)_2_. In the PN and PS samples after 56 days of electric pulse treatment, the characteristic peaks of Mg(OH)_2_ were absent, but multiple diffraction peaks of MgSiO_3_ were present. This suggests that the active SiO_2_ in NSP and SF combined with Mg(OH)_2_ in the weakly alkaline environment at the cathode end to form MgSiO_3_, resulting in less Mg(OH)_2_ precipitation in PN and PS samples, which was not detected by XRD.

**Figure 8:**
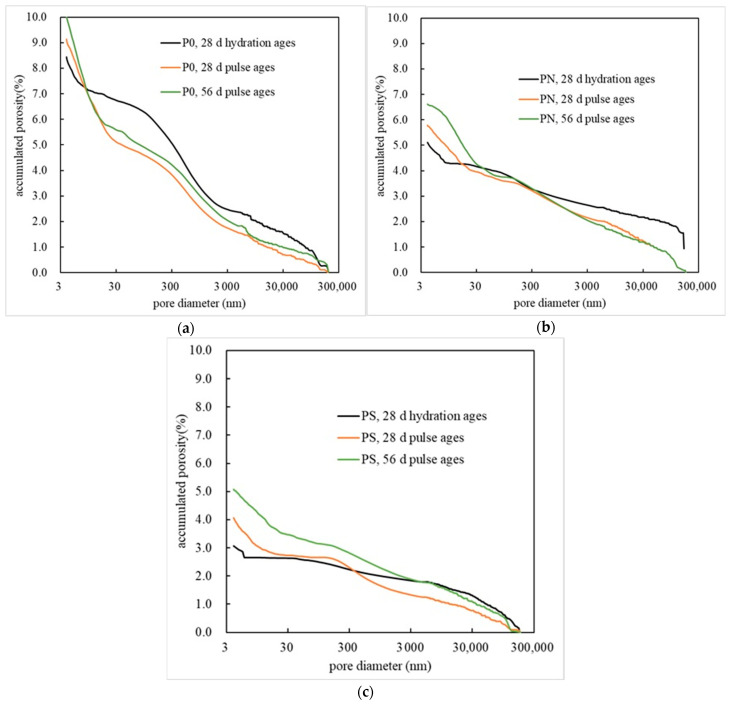
The accumulated porosity of MKPC specimens: (**a**) P0; (**b**) PN; (**c**) PS.

Figure 8 shows the cumulative pore volume of PMPC samples, obtained through MIP analysis, as pore size changes. The pore structure parameters are shown in Table 5. From Figure 8 and Table 6, it can be seen that after 28 days of hydration, the order of total porosity, from largest to smallest, for the three different PMPC samples is as follows: P0 (8.4441%), PN (5.1131%), and PS (3.0582%). Pulse corrosion gradually increases the total porosity of the three PMPC samples. When the pulse corrosion age is 56 days, the order of total porosity, from largest to smallest, for the three different samples is as follows: P0 (9.9977%), PN (5.7905%), and PS (5.0793%). According to Table 6, the total porosity of the three PMPC samples gradually increases with the extension of the corrosion age, which is attributed to the adsorption of K^+^ and Mg^2+^ from the pore solution of the PMPC matrix by the cathode electrode, leading to the dissociation and erosion of MKP within the PMPC slurry. As the corrosion age extends, the proportion of harmful pores less than 50 nm in the three PMPC samples increases synchronously with the total porosity, while the proportion of harmful pores greater than 200 nm, and between 50 and 200 nm gradually decreases. This is due to the precipitation of MgSO_4_·7H_2_O and Mg(OH)_2_ when SO_4_^2−^ and OH^−^ in the pore solution of the PMPC slurry reach saturation concentration, which can fill the large harmful pores. Additionally, the active SiO_2_ in NSP and SF combines with Mg(OH)_2_ in the weakly alkaline environment at the cathode end to form MgSiO_3_ gel, which can also fill the harmful pores in the matrix.

## 4. Discussion

**Figure 9:**
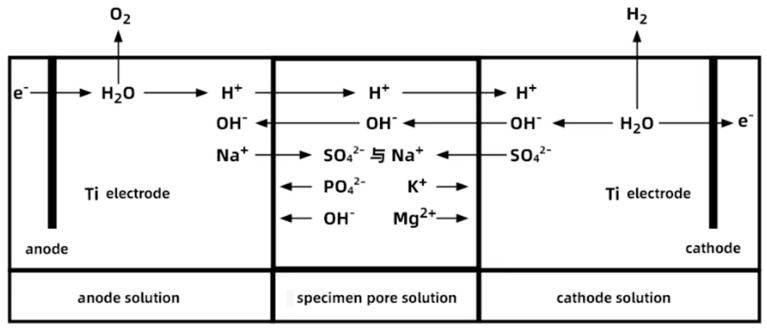
The ionic movement and reaction in the solution at both electrodes, and pore solution at the specimen.

The schematic diagram in Figure 9 reflects the ion movement and chemical reactions in the electrode solution under pulse two. The reason for the bubble formation and pH increase in the cathode solution at the initial stage of the electric pulse is that the electric pulse causes a reduction reaction at the cathode, producing hydrogen gas (2H^+^ + 2e^−^ → H_2_↑), and leading to a higher OH^−^ concentration than H^+^ in the solution. Under the electric field, the main ions (OH^−^ and SO_4_^2−^) migrate into the specimen through its open pores. The reason for the bubble formation and pH decrease in the anode solution at the initial stage of the electric pulse is that the pulse causes OH^−^ in the anode solution to lose electrons and release oxygen (4OH^−^ − 4e^−^ → 2H_2_O + O_2_↑), gradually reducing the OH^−^ concentration and lowering the pH value. Under the electric field, the main ions (H^+^ and Na^+^) migrate into the specimen through its open pores. In a water-saturated state, K^+^ and Mg^2+^ in the pore solution of the PMPC specimen tend to move towards the cathode under the electric field, while OH^−^ and PO_4_^3−^ tend to move towards the anode.

With regard to the PMPC specimens subjected to electro-pulse corrosion, during the initial stage of pulse corrosion, SO_4_^2−^ ions migrate into the interior of the cathode-end specimen under the dual influence of the electric field and concentration gradient (as shown in Figure 9 schematic). The migration ability of SO_4_^2−^ is significantly higher than that of fully immersed specimens driven solely by concentration gradients [28,29]. As SO_4_^2−^ passes through the pores of the PMPC specimen, the effect of the pulsed electric field on the pore structure surface frequently adjusts the direction of force and movement of the ions, producing a vibration effect similar to sieving materials, allowing SO_4_^2−^ to more effectively enter and pass through the small pores and fine gaps in the PMPC paste. With the extension of the pulse corrosion time, the cathode-end pulse electrode adsorbs K^+^ and Mg^2+^ from the pore solution of the PMPC matrix (as shown in Figure 9 schematic), leading to the dissociation and erosion of the hydration product MgKPO_4_·6H_2_O (MKP) within the hardened body [33,34,35]. When SO_4_^2−^ and OH^−^ migrate to the pore solution of the PMPC specimen from the cathode end and reach saturation concentration (as shown in Figure 9 schematic), low solubility MgSO_4_·7H_2_O and Mg(OH)_2_ precipitate [35,36]. The dissociation and erosion of MKP results in the degradation of the pore structure of the PMPC slurry (Table 6) [33,34,35]. However, due to the short corrosion cycle (≤56 days) and the filling effect of MgSO_4_·7H_2_O and Mg(OH) _2_, the degree of degradation of the pore structure at the cathode end of the pulse-corroded PMPC specimen is significantly less than that of fully immersed specimens [28,29]. But under the push of the electric field and vibration effect, more SO_4_^2−^ enters the deeper part of the PMPC matrix at the cathode end, and its actual measured migration depth (h_0_) is significantly greater than that of fully immersed PMPC specimens [28,29]. 

The addition of an appropriate amount of NSP or SF with a smaller average particle size can improve the particle grading of MgO powder, reducing the ratio of water to binder of the PMPC slurry with the same fluidity (Table 2) and decreasing the porosity formed through evaporating excess water when hydration (Table 6). As hydration progresses, the pH value of the PMPC slurry gradually increases, and the activity of the vitreous in NSP is activated in a weakly alkaline solution environment, producing a second hydration reaction. The generated MgSiO_3_ gel and Fe_3_(OH)_3_(PO_4_)_2_ further fill the pores and cracks of the PMPC slurry (Figure 7a) [37]. Under the effect of the hydration heat of the PMPC slurry, the active SiO_2_ in SF reacts with MgO to form MgSiO_3_ gel [38], which fills the pores in the hardened slurry. The aforementioned physical and chemical effects of NSP and SF improve the structure of the PMPC paste (Table 6), increasing the 28-day strength of PN and PS specimens compared to the reference specimen P0 (Table 2) [37,38]. In an electro-pulse corrosion environment, as the corrosion age extends, the activity of the glass phase in NSP is still activated under weakly alkaline solution conditions at the cathode end, triggering secondary hydration reactions and producing hydration products, such as MgSiO_3_ gel (Figure 7b), which further fill the pores and cracks of the PN slurry and improve the structure of the PN slurry (see Figure 8 and Table 6). The active SiO_2_ in SF combines with Mg^2+^ and OH^−^ under weakly alkaline solution conditions at the cathode end to form MgSiO_3_ gel (Figure 7b), which further fill the pores and cracks of the PS slurry and improve the structure of the PS slurry (see Figure 8 and Table 6). These effects can obstruct the SO_4_^2−^ migration in the PMPC slurry.

The effect of the pulsed electric field causes the SO_4_^2−^ on the surface structure of the PMPC specimen to frequently adjust its force and direction of movement, allowing SO_4_^2−^ to more effectively enter and pass through the micro-pores and fine gaps in the PMPC slurry. A small amount of sulfate (sulfate content < 0.01%) can migrate deeper, through the gaps in the matrix, resulting in h_0_ > h_00_. As the corrosion age extends, the pulsed cathode end will adsorb K^+^ and Mg^2+^ from the matrix pore solution, causing the MKP in the slurry to undergo dissolution and phase transformation [33,34,35]. The process above leads to the deterioration of the PMPC matrix’s pore structure (see Table 6) and increases the migration channels for SO_4_^2−^ into the slurry, resulting in the gradual increase in the h_00_. In the initial stage of electro-pulse corrosion of the PMPC specimen, under the dual effects of concentration difference and electric field, the migration speed of SO_4_^2−^ from the cathode end solution into the PMPC specimen is fast, with D being the maximum. As the corrosion age extends, the SO_4_^2−^ and OH^−^ in the specimen pore solution reach saturation concentration, producing precipitates of MgSO_4_·7H_2_O and Mg(OH)_2_, which can fill the pores in the PMPC slurry, slowing down the SO_4_^2−^ migration speed into the PMPC specimens, and gradually decreasing D. The initial porosity of specimens PN and PS is less than that of specimen P0 (Table 6), resulting in both the h_00_ and D for the SO_4_^2−^ migration from cathode into the PMPC specimen being smaller than those of specimen P0. As the pulse corrosion age extends, the secondary hydration reaction of NSP generates hydration products, such as MgSiO_3_ gel, filling the pores and cracks in specimen PN. The active SiO_2_ reacts with Mg^2+^ and OH^−^ in weakly alkaline solutions to form MgSiO_3_ gel, filling the pores and cracks in specimen PS. These actions decrease the proportion of harmful pores in the specimens PN and PS (see Figure 8 and Table 6), resulting in their h_00_ and D being smaller than those of specimen P0 at various corrosion ages.

In the initial stage of pulse corrosion, the cathodic pulse electrode adsorbs K^+^ and Mg^2+^ from the pore solution of the PMPC matrix, which causes the dissociation and erosion of the MKP inside the PMPC slurry, resulting in the structural deterioration of the PMPC specimens (Table 6) and a decrease in compressive strength. The initial porosity of specimens PN and PS is lower than that of specimen P0, and the degree of dissociation and erosion of MKP inside the slurry is lighter, resulting in a smaller decrease in strength compared to specimen P0. As the pulse corrosion time extends, SO_4_^2−^ and OH^−^ migrate into the pore solution of the specimens from the cathode end and precipitate MgSO_4_·7H_2_O and Mg(OH)_2_ when they reach saturation concentration. This filling effect reduces the harmful pore content of the PMPC specimens (Table 6), increasing their strength. The formation of MgSiO_3_ gel in specimens PN and PS further reduces the proportion of harmful pores in the slurry (see Figure 8 and Table 6), resulting in a greater increase in strength compared to specimen P0.

## 5. Conclusions

This study achieved an accelerated corrosion of PMPC slurry specimens through the application of electric pulses. By comparing the sulfate diffusion behavior of different PMPC specimens, the resistance of PMPC slurry to sulfate erosion was evaluated. The conclusions are as follows:

(a) A basic test and analysis cycle of 14 days can be used to implement electro-pulse-accelerated sulfate ion migration on PMPC specimens. The *c* (x, t) in PMPC specimens with the same corrosion period rapidly decreases with increasing distance from the surface (x), and, under the condition of R^2^ ≥ 0.999, it conforms to a third-order polynomial rule. When the electro-pulse period was 56 days, the *c* (0, 56 d) and *h*_0_ for PN and PS were lower than that of reference P0. A moderate amount of NSP or SF can significantly strengthen the SO_4_^2−^ resistance of the PMPC matrix.
(b) The calculated migration depth *h*_00_ of the PMPC specimens at each corrosion age is greater than *h*_0_. The migration coefficient *D* gradually decreases as the corrosion age extends. After 56 days of electro-pulse corrosion, the *h*_00_ and *D* of specimens PN and PS were lower than that of reference P0. The *D* values for PMPC specimens were one order of magnitude higher than those of PMPC specimens fully immersed for 360 days. The electro-pulse effect can significantly accelerate the SO_4_^2−^ migration in the PMPC specimens. A moderate amount of NSP or SF can significantly reduce the migration parameters *h*_00_ and D of the PMPC specimens.
(c) PMPC specimens subjected to pulse corrosion exhibited a logarithmic relationship between their corrosion age t and the calculated migration depth *h*_00_, and the sulfate migration coefficient *D*, with a correlation coefficient R^2^ > 0.98. The *h*_00_ and *D* could be estimated based on *t* due to the logarithmic relationship. Therefore, the sulfate resistance of PMPC specimens can be evaluated through short-term accelerated corrosion tests using electrical pulses. The research findings provide a theoretical foundation for the application and quality evaluation of PMPC-based material.
(d) When the corrosion age is 0, the order of specimen strengths, from smallest to largest, is P0, PN and PS. After 14 days of pulse corrosion, the compressive strength of all specimens slightly decreased compared to the initial strength. As the corrosion age extends further, the strength of PMPC specimens gradually increases. By 56 days of corrosion age, the increase rates in strength were 7.14% for P0, 7.94% for PN and 8.42% for PS.

## Figures and Tables

**Table 1:** Chemical components of MP, NSP, and SF.

Chemical Composition		MgO	SiO_2_	CaO	Fe_2_O_3_	Al_2_O_3_	CO_3_	Loi	Others
Content/%	MP	91.85	3.68	2.14	0.86	0.17		1.01	0.29
	NSP	31.28	48.65	1.35	8.05	3.41	5.73	1.02	0.51
	SF	0.05	97.42	0.04	0.02	0.05		2.26	0.16

**Table 2:** Mix proportion, flowability, and strengths of PMPC slurry.

Code Name	*m*_base_ /*m*_acid_	*m*_NSP or SF_ */m*_base_	*m*_CR_/ *m*_base_	W/C	Fluidity (mm)	FS (MPa)		CS (MPa)	
						3 d	28 d	3 d	28 d
P0	3:1	0	0.13	0.115	165	8.0	12.4	48.6	62.1
PN		0.2 NSP		0.108	163	8.6	12.9	52.4	66.2
PS		0.1 SF		0.114	161	8.7	13.1	56.7	69.7

FS—flexural strength; CS—compressive strength.

**Table 3:** The project, instrument, and analysis conditions for microanalysis of samples.

Sample Status	Project	Instrument	Analysis Conditions
powder	XRD	X-ray diffraction analyzer, D/max-RB, Rigaku, Tokyo, Japan	scanning range of 5–80°, and a scanning speed of 10°/min
small piece	MIP	fully automatic porosity analyser, PoreMaster-60, Boynton Beach, FL, USA	low pressure 55 psi and high pressure 40,000 psi

**Table 4:** Sulfate Ion Migration Parameters in PMPC specimens.

Cade	*t* (d)	*c* (0, t) (%)	*c* (*h*_00_, *t*) (%)	*h*_0_ (mm)	*h*_00_ (mm)	*D* (×10^−6^)
P0	14	0.1627	0.0069	14	12	14.32
	28	0.2831	0.0029	18	17	9.12
	42	0.3528	0.0028	22	20	7.80
	56	0.3959	0.0024	24	21	6.18
PN	14	0.1517	0.0001	14	11	11.02
	28	0.2650	0.0018	18	15	6.31
	42	0.3310	0.0014	20	17	5.81
	56	0.3672	0.0012	22	20	4.78
PS	14	0.1140	0.0019	12	10	9.31
	28	0.2209	0.0020	14	13	5.16
	42	0.2895	0.0010	18	15	3.62
	56	0.3325	0.0010	20	17	3.42

**Table 5:** The relationship model between t and h_00_, D.

Specimen Code	Computation Model	Correlation Coefficient (R^2^)
P0	*h*_00_ = 6.6887ln*t* − 5.4663	0.989
PN	*h*_00_ = 6.2215ln*t* − 5.6112	0.981
PS	*h*_00_ = 4.9427ln*t* − 3.2209	0.990
P0	D = 66.821 t^0.587^	0.993
PN	D = 49.124 t^0.583^	0.966
PS	D = 66.295 t^0.756^	0.989

**Table 6:** The pore structure parameters for PMPC samples.

Code	Curing/Corrosion Condition	Total Porosity /%	Pore Volume Distribution/%		
			<50 nm	50–200 nm	>200 nm
P0	28 d hydration ages	8.4441	21.85	12.45	65.70
	28 d pulse ages	9.1410	46.56	8.19	45.28
	56 d pulse ages	9.9977	47.00	8.13	44.87
PN	28 d hydration ages	5.1131	15.75	4.67	79.58
	28 d pulse ages	5.7905	34.76	6.6	58.64
	56 d pulse ages	6.6178	40.36	6.77	52.87
PS	28 d hydration ages	3.0582	15.75	8.27	75.98
	28 d pulse ages	4.0553	33.7	5.1	61.20
	56 d pulse ages	5.0793	34.76	6.64	58.60

## Data Availability

The original contributions presented in this study are included in the article. Further inquiries can be directed to the corresponding author.
